# Supplementary material for: Increasing the density of passive photonic-integrated circuits via nanophotonic cloaking
Source: Nat Commun. 2016 Nov 9;7:13126. doi: 10.1038/ncomms13126 (PMC5105174; doi:10.1038/ncomms13126)
Supplement: Supplementary Information — Supplementary Figures 1-5, Supplementary Note 1 and Supplementary Methods. [file ncomms13126-s1.pdf]

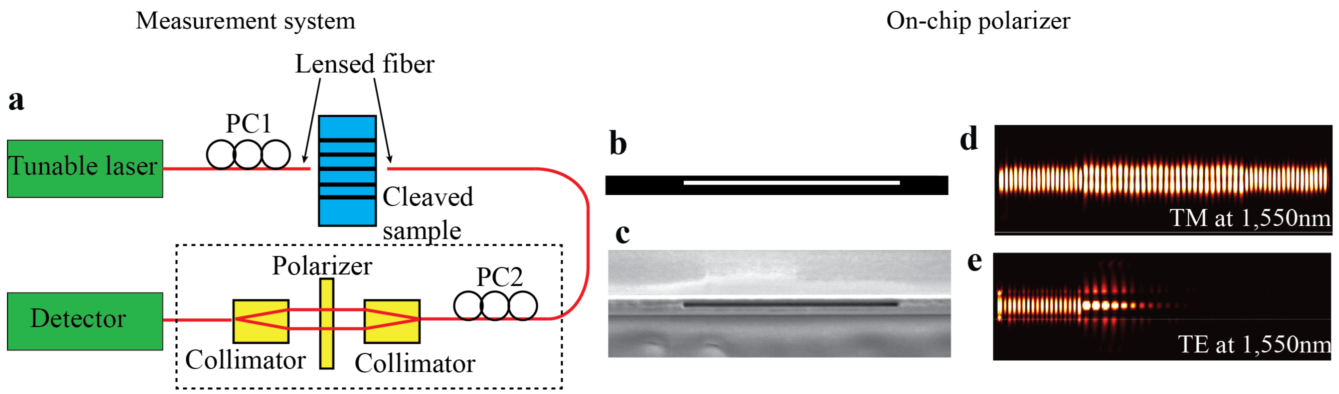

Supplementary Figure 1: Characterization. (a) Measurement system. (b) Design and (c) Scanning-electron micrographs (SEM) of the on-chip polarizer. Steady-state field intensity pattern of (d) TM and (e) TE mode for the on-chip polarizer. The on-chip polarizer is used to select the particular polarization state launched in the device.

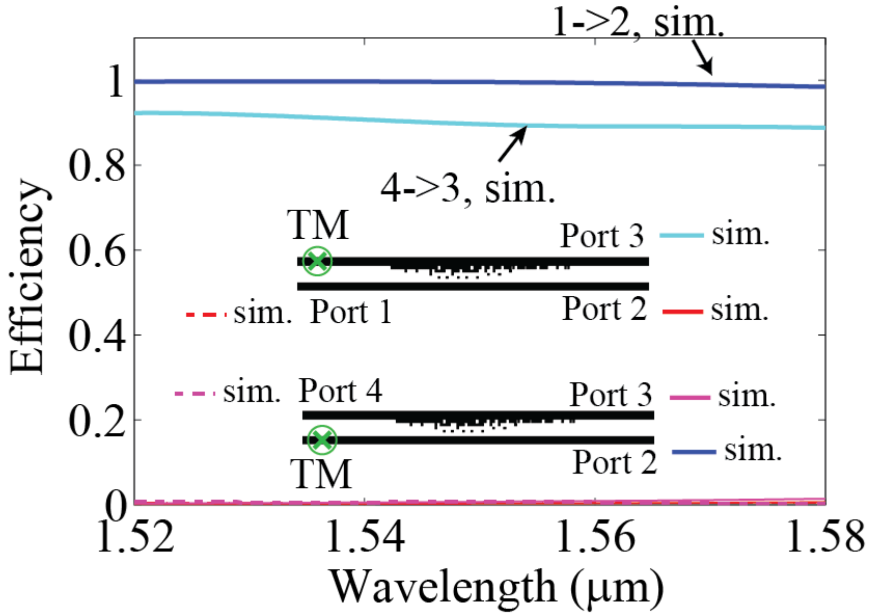

Supplementary Figure 2: Transmission efficiencies for experimental TM-cloak. The transmission efficiencies for port 1 to port 2, port 1 to port 4 and port 1 to port 3 are the same with those in Fig. 2 in the manuscript. The simulated and measured transmission efficiencies for port 4 to port 3 are represented by cyan solid and red dotted curve, respectively. It is confirmed that the cloak is symmetric for the parallel identical waveguides under TM illumination.

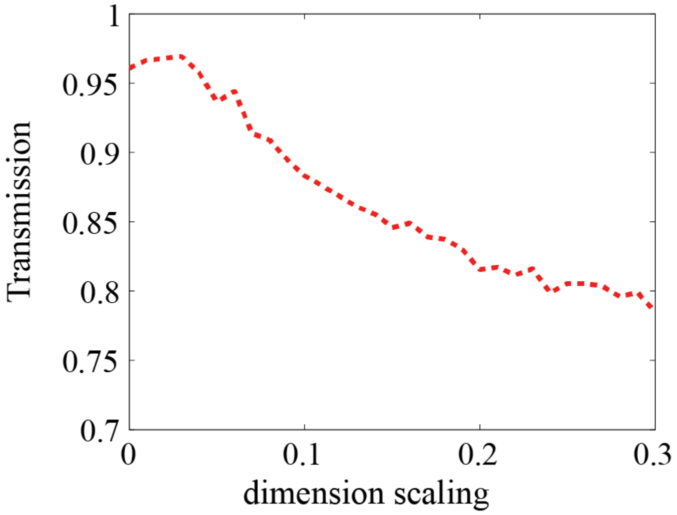

Supplementary Figure 3: Scaling-error analysis of device from Fig. 4(a). We performed a numerical analysis of the transmission efficiency of the device from Fig. 4(a) in the main text. The analysis was performed by scaling the geometry of the device. From this analysis, we can see that in order to maintain transmission efficiency >90%, we need to control the dimension of the device to about 12nm.

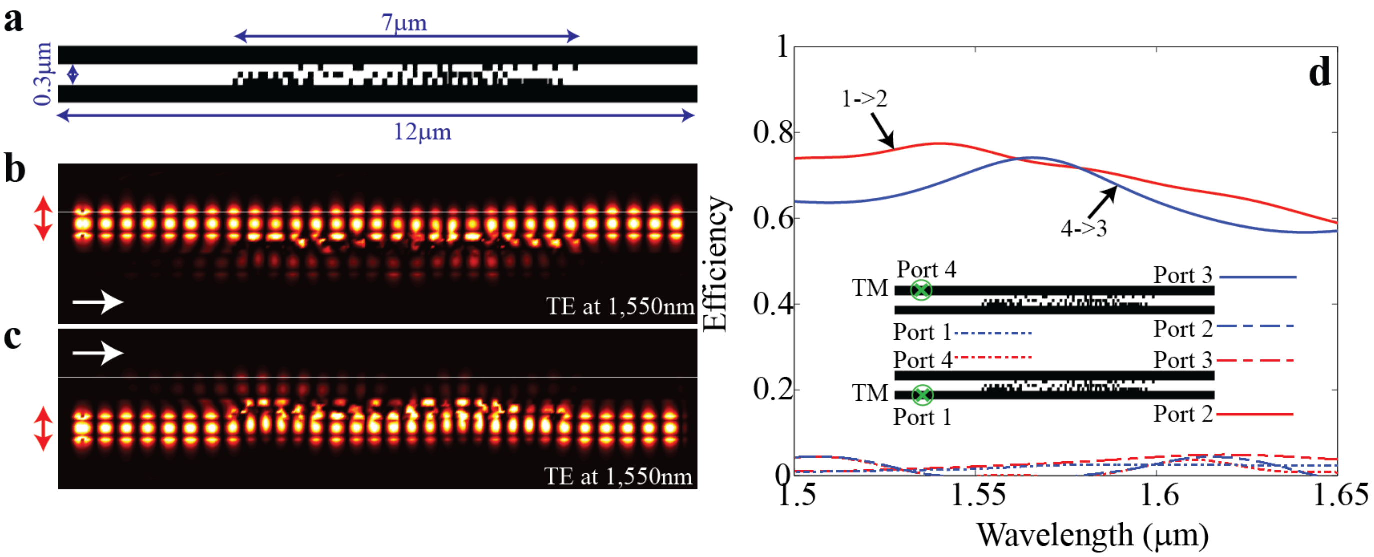

Supplementary Figure 4: Minimal spacing between neighboring waveguides with cloak. (a) The geometry of the TE-cloak with centre-to-centre spacing of 0.6 μm. The simulated steady-state intensity distribution at λ_0=1550nm are shown for (b) TE and input at port 4, and (c) TE and input at port 1. (d) The simulated efficiencies for TE-cloak with center-to-center spacing of 0.6 μm.

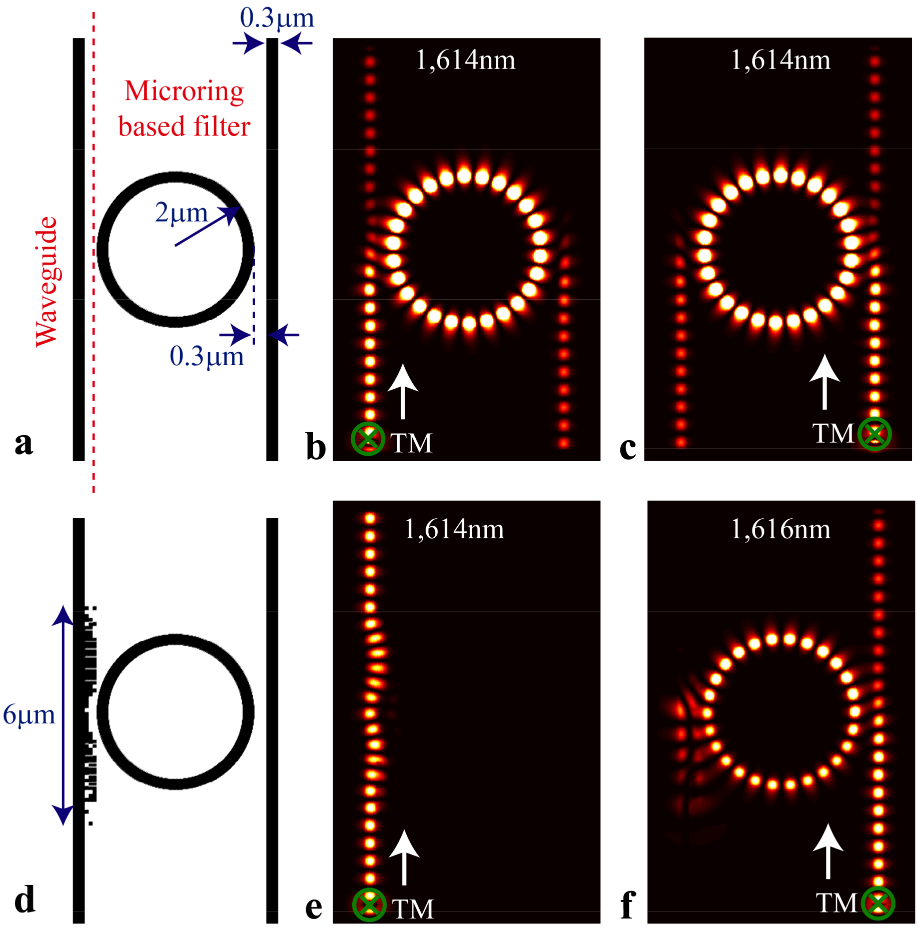

Supplementary Figure 5: Cloak for microring resonator. (a) Epsilon distribution of the reference coupled system composed of a waveguide and a microring based filter. Steady-state electric intensity pattern for the coupled system in (a) when TM source is launched in the (b) left and (c) right waveguide. (d) Epsilon distribution of a system composed of a waveguide, a cloak and a microring based filter. Steady-state electric intensity pattern for the coupled system in (d) when TM source is launched in the (e) left and (f) right waveguide. Although the cloak is designed to hide the ring from the left waveguide, the cloak is also symmetric, which means that the left waveguide could also be hidden from the ring, as mentioned in the manuscript. The waveguide and the microring based filter works independently as if the two components are invisible to each other.

Supplementary Note 1. 0.3μm spacing between parallel waveguides

We now squeeze the spacing between neighboring waveguides even further to check the limit of the waveguide cloak. For TM mode, large cross-talk is inevitable below the center-to-center spacing of 0.7 μm. The spacing for TE case could be further squeezed down to 0.6 μm without significantly sacrificing the cross-talk. The results of the TE-cloak are summarized in Supplementary Fig. 3. The centre-to-centre spacing between the neighbouring waveguides is 0.6 μm with a waveguide width of 0.3 μm as shown in Supplementary Fig. 3(a). The steady-state electric field intensity patterns as shown in Supplementary Fig. 3(b) and 3(c) reveal that the energy could be well confined in the corresponding waveguide with negligible energy coupled to the neighboring waveguide. The simulated transmission efficiency at 1550nm is 71.2% and 76.4% for signal launched in the top (port 4 to port 3) and bottom (port 1 to port 2) waveguide, respectively. The crosstalk for both case, on the other hand, is less than 1.5%, which corresponds to an extinction ratio larger than 16.8dB and 17.1dB for the two waveguides.

Supplementary Methods

$ÅÔÁÉÌÅÄ ÓÁÍÐÌÅ ÐÒÅÐÁÒÁÔÉÏÎ ÍÅÔÈÏÄ

Our device is fabricated on a silicon-on-insulator (SOI) wafer with a device thickness of 300nm. Two-step fabrication process is adopted since we do not have access to a lithography tool with required resolution for an exposing area in the order of millimeters (e.g. electron-beam lithography), although our device can be fabricated via a single-lithography step. First, the SOI wafer is coated by a thin layer of HDMS before spin coating the photoresist – Shiply 1813. Photolithography tool (Heidelberg μPG 101) is then used to generate the pattern for the multi-mode waveguide interfacing lensed fiber and tapper on the photoresist after the coating process. The sample is then immersed in the developer AZ 1:1 for one minute that is followed by being immersed in DI wafer for two minutes to stop the developing. The pattern on the photoresist is then transferred to the top silicon layer of SOI via deep reactive-ion etcher (Oxford 100) with a mixture etching gas of SF_6 and C_4F_8 with a flow rate of 40ccm and 17.5ccm, respectively. Second, we use dual-beam focued-ion beam (FEI Helios 650) to define the fine cloak region with feature size 100nm. The beam current used was 7.7pA with fluence of 800C/m^2. Alignment were used to correct the systematic beam drift caused by the change of surrounding temperature, moisture and so on. We also fabricated reference devices including the same tapers as the PBS devices for normalization, and an on-chip polarizer for polarization state alignment.

#ÈÁÒÁÃÔÅÒÉÚÁÔÉÏÎ ÍÅÔÈÏÄ

The characterization is summarized in Supplementary Figure 1. Supplementary Fig. 1(a) illustrates the measurement system and Supplementary Figs. 1(B)- 1(E) show the on-chip polarizer used in characterization. The on-chip polarizer employed here is a pretty straightforward one that is consist of a straight waveguide with a vertical air slot near the center of the waveguide. The center of the air slot exhibits a 70nm offset with regard to the center of the waveguide. The detailed characterization process is as follows.

First, we bypass the optical components within the dotted frame by connecting the lensed fiber in the output path to the detector directly. The lensed fiber in the input path is moved to the on-chip polarizer. The polarization state of the input light is selected by rotating the polarization controller 1 (PC1) and monitoring the received power. The on-chip polarizer allows the TM mode to pass through efficiently while blocking the light of the orthogonal polarization state as illustrated in Supplementary Figs. 1(d)- 1(e). As a result, the TM input mode is selected by maximizing the power detected and TE is selected by minimizing the power.

Second, the input lensed fiber is moved to a straight waveguide without any pattern on the sample and the optical components within the dotted frame is inserted to the output path. We rotate the polarization controller 2 (PC2) in the output path to align the polarization plane of the output light with that of the polarizer.

Third, we move the input lensed fiber to the cloak. The polarization components of the output light is measured via rotating the polarizer correspondingly.

For measuring the transmission spectrum of light ending at the intended port, the measurement agrees well with the simulation, as shown in Fig. 2 in the manuscript. However, measurement of the cross-talk is a little bit trick. For our case, the cross-talk is typically around 0.1% and the coupling efficiency between the fiber and the waveguide is estimated to be 10%. The source power after the fiber is around 1mW and the transmission efficiency after the collimator-collimator pair is around 10%. As a result, the typical power for the cross-talk for our measurement system is 1nW. However, the minimal detectable power of the diode is 0.1nW, which is pretty close to signal level. As a result, we could not obtain a reliable measurement and measurement data with error bar is used to give a general idea of the cross talk. The next step is to increase the coupling efficiency in the various interfaces, especially the coupling efficiency between the lensed fiber and waveguide. The cross-talk could be reliably measured only if the signal level is sufficiently large.

Upgraded design algorithm

As mentioned earlier, direct-binary search (DBS) optimization algorithm coupled with finite-difference time-domain (FDTD) method was previously employed in designing our all-dielectric nanophotonic cloak. The DBS algorithm is tentative to converges to a local maximum and the possibility of encountering the global maximum is increased by careful selecting the starting point and running multiple independent optimizations simultaneously. For our experimental designs, 10 independent optimizations were ran simultaneously and we selected the design with the highest efficiency for experimental verification. The ~90% transmission efficiency is acceptable to demonstrate the proof-of-principle. In order to further decrease the insertion loss to warrant its applications in long-range energy transportation, Particle Swarm Optimization (PSO) is incorporated in the design algorithm to explore the limit of the insertion loss. PSO was first proposed by Eberhart *et al.* through simulating the social behavior of flying birds. Each individual, called a particle, adjusts its flight according to both its own and its neighbor' flying experiences. The position of a particle is updated via the following equation,

$$x_{i,d} = x_{i,d} + v_{i,d} \cdot \Delta t \tag{1}$$

$$v_{i,d} = \varpi_n \cdot v_{i,d} + c_1 \cdot rand() \cdot \left(p_{i,d} - x_{i,d} \right) + c_2 \cdot rand() \cdot \left(g_{i,d} - x_{i,d} \right) \tag{2}$$

where $x_{i,d}$ is the ith particle's position in the dth dimension of the parameter space, and $v_{i,d}$ is the corresponding velocity. w_n is the inertial weight for n^{th} iteration and determines how likely the particle stays on its old velocity. $p_{i,d}$ and $g_{i,d}$ are individual and global best positions, respectively. c_1 and c_2 are two positive constants, and determine how much a particle is influenced by the memory of its own best position and the global best position, respectively. For our case, a large inertial weight is used to traverse most of the design space and finally a smaller inertial weight is employed for convergence. As DBS and PSO exhibits the distinct properties, that is DBS tends to converge to local maximum with a few iterations and PSO is much more likely to converge to the global maximum but with several hundreds of iterations, we first use DBS to quickly arrive at a local maximum, which serves as the initial position of one of the particles for PSO to further improve the design. It is found that PSO typically converges with less than 20 iterations, much faster than optimization solely based on PSO that typically exhibits hundreds of iterations. In addition, geometry constraint is applied to the design as well. It is found blocks with multiple silicon pillars connected to each other is far more immune from fabrication errors than individual silicon pillars due to the rounded corners. To generate a design facilitating fabrication, weighing factor is introduced to enable the algorithm to arrive at a design with few isolated silicon pillars and thus higher robustness to fabrication errors.
